# Comorbidities of psychiatric and headache disorders in Nepal: implications from a nationwide population-based study

**DOI:** 10.1186/s10194-016-0635-8

**Published:** 2016-04-22

**Authors:** Ajay Risal, Kedar Manandhar, Are Holen, Timothy J. Steiner, Mattias Linde

**Affiliations:** Department of Neuroscience, Norwegian University of Science and Technology, Nevrosenteret Øst, St Olavs Hospital, 7006 Trondheim, Norway; Dhulikhel Hospital, Kathmandu University Hospital, Dhulikhel, Kavre Nepal; Pain Unit, St Olavs University Hospital, Trondheim, Norway; Division of Brain Sciences, Imperial College London, London, UK; Norwegian Advisory Unit on Headache, St Olavs University Hospital, Trondheim, Norway

**Keywords:** Anxiety, Comorbidity, Depression, Headache disorders, Holistic care, Nepal, Neuroticism, Primary care, Questionnaire, South Asia, Global campaign against headache

## Abstract

**Background:**

Headache disorders, anxiety and depression – the major disorders of the brain – are highly comorbid in the western world. Whether this is so in South Asia has not been investigated, but the question is of public-health importance to countries in the region. We aimed to investigate associations, and their direction(s), between headache disorders (migraine, tension-type headache [TTH] and headache on ≥15 days/month) and psychiatric manifestations (anxiety, depression and neuroticism), and how these might affect quality of life (QoL).

**Methods:**

In a nationwide, cross-sectional survey of the adult Nepalese population (*N* = 2100), trained interviewers applied: 1) a culturally-adapted version of the *Headache-Attributed Restriction, Disability, Social Handicap and Impaired Participation* (HARDSHIP) questionnaire to diagnose headache disorders; 2) a validated Nepali version of the *Hospital Anxiety and Depression Scale* (HADS) to detect anxiety (HADS-A), depression (HADS-D) and comorbid anxiety and depression (HADS-cAD); 3) a validated Nepali version of the *Eysenck Personality Questionnaire Revised Short Form-Neuroticism* (EPQRS-N); and 4) the *World Health Organization Quality of Life 8-question scale* (WHOQOL-8). Associations with headache types were analysed using logistic regression for psychiatric caseness and linear regression for neuroticism. Adjustments were made for age, gender, household consumption, habitat, altitude and use of alcohol and marijuana.

**Results:**

HADS-A was associated with any headache (*p* = 0.024), most strongly headache on ≥15 days/month (AOR = 3.2) followed by migraine (AOR = 1.7). HADS-cAD was also associated with any headache (*p* = 0.050, more strongly among females than males [*p* = 0.047]) and again most strongly with headache on ≥15 days/month (AOR = 2.7), then migraine (AOR = 2.3). Likewise, neuroticism was associated with any headache (*p* < 0.001), most strongly with headache on ≥15 days/month (B = 1.6), followed by migraine (B = 1.3). No associations were found between HADS-D and any headache type, or between TTH and any psychiatric manifestation. Psychiatric caseness of any sort, when comorbid with migraine or TTH, aggravated the negative impact on QoL (*p* < 0.001).

**Conclusion:**

Headache disorders are highly comorbid with anxiety and show associations with neuroticism in Nepal, with negative consequences for QoL. These findings call for reciprocal awareness, and a holistic coordinated approach to management and in the health service. Care for common headache and common psychiatric disorders should be integrated in primary care.

## Background

The term “comorbidity” refers to the coexistence of any additional ailment in a person with an index disease [1]. Headache disorders such as migraine and tension-type headache (TTH) and psychiatric disorders such as anxiety and depression are all very common among general populations worldwide. Accordingly, some level of comorbidity between them will arise by chance. However, epidemiological studies over 25 years have consistently indicated that headache disorders and anxiety and depression are excessively comorbid [2–20], with bidirectional [6] or syndromal [7] associations. Although most such studies have selectively considered migraine [8–11], some have looked at TTH [12], “chronic daily headache” (CDH) [13] or headache in general [14]. Furthermore there are studies showing associations between headache disorders and neuroticism [21, 22], a personality trait closely interlinked with psychological distress [23].

Headache disorders, anxiety and depression are collectively viewed as the major disorders of the brain (MDBs) [24], each occupying a place among the top ten causes of disability in the world [25, 26]. From a public-health perspective, worrying consequences for overall disease burden arise from these disorders being comorbid. Firstly, comorbidity increases morbidity, perhaps synergistically. For example, headache associated with psychopathology has exaggerated effects on quality of life (QoL) and disability [23, 27, 28]. Secondly, comorbid disorders may be mutually aggravating. Again for example, comorbid psychiatric illnesses are risk factors for headache becoming chronic [29]. Thirdly, comorbid headache and psychiatric disorders pose significant management challenges: treatment of each may be hindered by the other, with worse outcomes likely and increased health-care liabilities [30]. Fourthly, there is the possibility of causal relationships, in either or both directions.

Most data on this subject come from the western world. However, MDBs appear to be prevalent and burdensome everywhere. The Global Burden of Disease Study 2013 (GBD2013) extrapolations to South Asia indicated that years of life lost to disability (YLDs) in this Region from these conditions were in line with global rankings [25]. Our study from Nepal, the only nationwide study in this region so far, showed high prevalences of both headache and psychiatric disorders, including a much higher prevalence of migraine than the global mean [31] and prevalences of anxiety above and of depression at the upper limit of their respective global ranges [32]. Both anxiety and depression correlated positively with neuroticism and negatively with QoL [32]. The probability of comorbidity between these disorders in Nepal, and the implications arising therefrom, are consequently matters of considerable public-health importance and of interest to health policy.

Therefore, our aims were to examine the extent to which the common headache disorders (migraine, TTH and the group of headache disorders characterised by headache occurring on ≥15 days/month) and psychiatric disorders (anxiety and depression) are comorbid in Nepal, a country with unique geocultural diversity [33], and to look also for associations between these headache disorders and neuroticism. Our objective was to establish the public-health implications of any associations discovered. Our purpose was to guide national health policy.

## Methods

### Ethics

This study was part of a research project addressing MDBs in Nepal [33], approved by the Nepal Health Research Council, the Institutional Review Committee of Kathmandu University School of Medical Sciences, Dhulikhel Hospital, and the Regional Committee for Health and Research Ethics in Central Norway. Informed consent was collected from all participants.

### Study design and sampling

This was a cross-sectional study in which unannounced household visits were made during May 2013 by trained interviewers using structured questionnaires. In order to obtain a representative sample of the adult general population of the country, we used a multistage random cluster sampling technique to select households in all three physiographic divisions of Nepal, and, within each division, all five development regions (Far-Western, Mid-Western, Western, Central and Eastern). From each household, we randomly selected one adult aged 18–65 years. The details of the sampling and data collection procedures, including the steps taken to ensure a very high participation rate, have been published elsewhere [34].

### Survey instrument

We used the Headache-Attributed Restriction, Disability, Social Handicap and Impaired Participation (HARDSHIP) questionnaire [35], translated and culturally adapted for Nepal. It included demographic enquiry followed by modules assessing headache, psychiatric comorbidities, neuroticism and QoL.

All parts of the instrument, including these modules, were interviewer-administered.

#### Headache enquiry

The screening question asked “Did you have headache in the last 12 months?” Those who answered “no” were classified as headache-free and served as the reference group. Those responding “yes” were asked a series of diagnostic questions based on the International Classification of Headache Disorders (ICHD-3 beta) [36]. Participants reporting more than one headache type were requested to focus only on the most bothersome type in response to these questions. Diagnoses were made algorithmically. Participants with headache on ≥15 days/month were first separated; those who were also overusing acute or symptomatic medication for headache were diagnosed as probable MOH (pMOH), and the remainder as “other headache on ≥15 days/month”. To all other participants, the algorithm applied ICHD-3 beta criteria, with modifications, in the order: definite migraine, definite TTH, probable migraine, probable TTH. Any remaining cases were unclassifiable. In the subsequent analyses, definite and probable migraine were considered together as migraine, and definite and probable TTH as TTH. This procedure, and the necessary adaptations made to certain of the ICHD-3 beta criteria, have been described in detail earlier [31].

#### Assessment of psychiatric comorbidities

Imported as a module into the HARDSHIP questionnaire was a validated Nepali version of the Hospital Anxiety and Depression Scale (HADS) [37]. This scale consists of 14 items in two subscales, HADS-Anxiety and HADS-Depression, each with seven items [38]. Each item expresses the subjective experience of the respondent in the preceding week, and is rated 0–3 (3 indicating maximum symptom severity) so that the sum of each subscale has a potential range of 0–21. In accordance with the original description of HADS [38] and our validation of the Nepali version [37], a threshold of 11 on the respective subscale was taken to indicate caseness for anxiety or depression. Participants scoring above the threshold on only one subscale were regarded as cases of anxiety (HADS-A) only or depression (HADS-D) only; those scoring above 11 on both were regarded as cases of comorbid anxiety and depression (HADS-cAD).

#### Assessment of neuroticism

Also imported as a module into the HARDSHIP questionnaire was a similarly validated version of the Eysenck Personality Questionnaire Revised Short Form-Neuroticism (EPQRS-N) [39]. It has 12 questions, each with the response options “no” (scored 0) and “yes” (scored 1) to assess the degree of neuroticism in a respondent [40]: the sum of responses has a potential range of 0–12, with higher values indicating more neuroticism.

#### Assessment of quality of life

Finally imported into the HARDSHIP questionnaire was a culturally-adapted version of the World Health Organization Quality-of-Life 8-question scale WHOQOL-8 [41]. This consists of eight questions addressing perceived aspects of the respondent’s QoL in four principal domains: psychological, physical, social and environmental. Each question has five response options on a Likert scale, scored from 1 (worst) to 5 (best); the summed score has the potential range of 8–40, with higher scores indicating better QoL.

### Statistical analysis

Analyses were carried out using IBM SPSS Statistics 21.

We used logistic regression analysis to examine associations between headache (all, and its different types: migraine, TTH or headache on ≥15 days/month) and psychiatric caseness (HADS-A, HADS-D or HADS-cAD). Both bivariate and multivariate analyses were undertaken with headache (or its types) as the independent variable; multivariate analyses were repeated with each of HADS-A, HADS-D and HADS-cAD as the independent variable. Odds ratios (ORs) with 95 % confidence intervals (CIs) are presented as the measure of association.

We used a general linear regression model (GLM) to identify associations between total neuroticism score and all headache or its types. The regression coefficient (B) represents the difference in neuroticism scores between participants with headache (or a headache type) and those with no headache (the reference category in both analyses).

In the logistic regression analyses as well as the GLM model, we adjusted for age (categorized 18–25, 26–35, 36–45, 46–55, 56–65 years), gender, annual household consumption (categorized ≤950, 950–1200, >1200 USD/year), habitat (urban *versus* rural), altitude (<2000 *versus* ≥2000 m) and the use of alcohol or marijuana (no *versus* yes). Potential interactions of headache types with demographic (age, gender) or environmental (habitat, altitude) factors in the associations with psychiatric caseness or total neuroticism score were tested by creating an interaction term (the product of the two independent variables: *eg*, age category*headache type) which was added to the regression model. If the interaction term reached statistical significance (*p* < 0.05), subgroup-specific results were derived.

We compared WHOQOL-8 scores among participants with each type of headache with or without psychiatric comorbidity, and among psychiatric cases with or without comorbid headache. We used Student’s t-test to compare differences in mean scores. We set *p* < 0.05 as the level of significance in all analyses.

## Results

There were 2100 participants, with a participation rate of 99.6 % (males: 861 [41.0 %]; females: 1239 [59.0 %]; mean age 36.4 ± 12.8 years). The majority (1328; 63.2 %) were rural inhabitants, and over one fifth (470; 22.4 %) lived at an altitude of ≥2000 m. Well over one third (822; 39.1 %) were in the lowest category of annual household consumption. A detailed description of the sample characteristics has been reported previously [34].

Having any headache was significantly associated with HADS-A caseness (17.2 %) compared with having no headache (10.5 %; AOR 1.6; 95 % CI: 1.1–2.3; *p* = 0.024) (Table 1). In relation to headache types, the strongest association with HADS-A was observed for headache on ≥15 days/month (AOR 3.2) followed by migraine (AOR 1.7); there was no significant association between TTH and HADS-A (Table 1). No interaction effects were seen with any of the factors analysed.

**Table 1:** Logistic regression analysis showing association of headache disorders with anxiety (HADS-A)

Headache type	HADS-A caseness			*p*
	*n* (%)^a^	OR [95 % CI]	AOR [95 % CI]	
No headache (*N* = 306)	32 (10.5)	Reference		
Any headache (*N* = 1794)	308 (17.2)	1.8 [1.2–2.6]	1.6 [1.1–2.3]	0.024
Migraine (*N* = 728)	134 (18.4)	1.9 [1.3–2.9]	1.7 [1.1–2.6]	0.013
Tension-type headache (*N* = 863)	121 (14.0)	1.4 [0.9–2.1]	1.3 [0.8–1.9]	0.29
Headache on ≥15 d/m (*N* = 161)	48 (29.8)	3.6 [2.2–5.9]	3.2 [1.9–5.4]	<0.001

^a^Number (n) of cases with anxiety (HADS-A) and their proportion (%) among those (N) with the headache type; OR: odds ratio, AOR: adjusted odds ratio (adjusted for age, gender, annual household consumption, habitation, altitude and use of alcohol and marijuana), d/m: days/month

There were no significant associations between having headache and HADS-D caseness (Table 2).

**Table 2:** Logistic regression analysis showing association of headache disorders with depression (HADS-D)

Headache type	HADS-D caseness			*p*
	*n* (%)^a^	OR [95 % CI]	AOR [95 % CI]	
No headache (*N* = 306)	15 (4.9)	Reference		
Any headache (*N* = 1794)	94 (5.2)	1.1 [0.6–1.9]	1.2 [0.6–2.0]	0.66
Migraine (*N* = 728)	44 (6.0)	1.3 [0.7–2.3]	1.3 [0.7–2.3]	0.49
Tension-type headache (*N* = 863)	36 (4.2)	0.8 [0.5–1.6]	0.9 [0.5–1.8]	0.91
Headache on ≥15 d/m (*N* = 161)	11 (6.8)	1.4 [0.6–3.2]	1.5 [0.6–3.4]	0.38

^a^Number (n) of cases with depression (HADS-D) and their proportion (%) among those (N) with the headache type; OR: odds ratio, AOR: adjusted odds ratio (adjusted for age, gender, annual household consumption, habitation, altitude and use of alcohol and marijuana), d/m: days/month

Having any headache was significantly associated with HADS-cAD caseness (7.0 %) compared with having no headache (3.6 %; AOR 1.9; 95 % CI: 1.0–3.6; *p* = 0.050) (Table 3). Again the strongest association with HADS-cAD was seen for headache on ≥15 days/month (AOR 2.7) followed by migraine (AOR 2.3), and there was no significant association between TTH and HADS-cAD.

**Table 3:** Logistic regression analysis showing association of headache disorders with comorbid anxiety and depression (HADS-cAD)

Headache type	HADS-cAD caseness			*p*
	*n* (%)^a^	OR [95 % CI]	AOR [95 % CI]	
No headache (*N* = 306)	11 (3.6)	Reference		
Any headache (*N* = 1794)	126 (7.0)	2.0 [1.1–3.8]	1.9 [1.0–3.6]	0.050
Migraine (*N* = 728)	64 (8.8)	2.6 [1.3–4.9]	2.3 [1.2–4.4]	0.016
Tension-type headache (*N* = 863)	42 (4.9)	1.4 [0.7–2.7]	1.4 [0.7–2.8]	0.34
Headache on ≥15 d/m (*N* = 161)	17 (10.6)	3.2 [1.4–6.9]	2.7 [1.2–6.1]	0.014

^a^Number (n) of cases with comorbid anxiety and depression (HADS-cAD) and their proportion (%) among those (N) with the headache type; OR: odds ratio, AOR: adjusted odds ratio (adjusted for age, gender, annual household consumption, habitation, altitude and use of alcohol and marijuana), d/m: days/month

In tests for interaction, we found only a weakly significant effect of gender (*p* = 0.047) with any headache. A subgroup analysis, however, suggested that the association of any headache with HADS-cAD was specific to females (AOR 4.3 [95 % CI: 1.3–13.8] *versus* AOR 1.0 [95 % CI: 0.4–2.2] in males). Numbers were low in the reference group in these analyses. No significant interactions were seen with any of the headache types.

Linear regression analysis showed a significant association between all headache and neuroticism score (B = 0.9; 95 % CI: 0.5–1.2; *p* < 0.001). Among headache types, headache on ≥15 days/month showed the strongest association (B = 1.6; 95 % CI: 0.9–2.2; *p* < 0.001), followed by migraine (B = 1.3; 95 % CI: 0.8–1.7; *p* < 0.001); the association between TTH and neuroticism was weak, although significant (B = 0.4; 95 % CI: 0.02–0.8; *p* = 0.040). All these analyses were adjusted for age, gender, household consumption, habitation, altitude and use of alcohol and marijuana. Tests for interaction revealed no significant effects with regard to any headache. However, in relation to migraine, the association with neuroticism demonstrated some gender-specificity, being stronger among males (B = 1.9; 95 % CI: 1.3–2.5) than females (B = 0.8; 95 % CI: 0.1–1.4).

Tables 4 and 5 summarise the data from Tables 1, 2 and 3, presenting them from a clinical management perspective: what was the probability (expressed as a percentage and AOR) that a patient with either headache (of a specific type) or psychiatric disorder (as HADS caseness) had the other as a comorbid condition? HADS-A was the most prevalent HADS caseness comorbid with each of the headache types: a patient with headache on ≥15 days/month had almost 30 % probability (AOR 3.2) of also having HADS-A. Migraine was the most prevalent headache comorbid with each of HADS-A, HADS-D and HADS-cAD: a patient with HADS-cAD had 46.7 % probability (AOR 2.5) of also having migraine.

**Table 4:** Prevalence of psychiatric disorder among participants with headache, by headache type

Headache type	HADS-A caseness (*N* = 340)		HADS-D caseness (*N* = 109)		HADS-cAD caseness (*N* = 137)	
	*n* (%)	AOR [95 % CI]	*n* (%)	AOR [95 % CI]	n (%)	AOR [95 % CI]
Migraine (*N* = 728)	134 (18.4)	1.7 [1.1–2.6]	44 (6.0)	1.3 [0.7–2.3]	64 (8.8)	2.3 [1.2–4.4]
Tension-type headache (*N* = 863)	121 (14.0)	1.3 [0.8–1.9]	36 (4.2)	0.9 [0.5–1.8]	42 (4.9)	1.4 [0.7–2.8]
Headache on ≥15 d/m (*N* = 161)	48 (29.8)	3.2 [1.9–5.4]	11 (6.8)	1.5 [0.6–3.4]	17 (10.6)	2.7 [1.2–6.1]

AOR: adjusted odds ratio (multivariate logistic regression analysis, using participants with no headache as reference and adjusted for age, gender, household consumption, habitation, altitude and use of alcohol and marijuana), d/m: days/month

**Table 5:** Prevalence of headache among participants with psychiatric disorder, by HADS caseness

HADS caseness	Migraine (*N* = 728)		Tension-type headache (*N* = 863)		Headache on ≥15 d/m (*N* = 161)	
	*n* (%)	AOR [95 % CI]	*n* (%)	AOR [95 % CI]	*n* (%)	AOR [95 % CI]
HADS-A (*N* = 340)	134 (39.4)	1.8 [1.2–2.7]	121 (35.6)	1.2 [0.8–1.8]	48 (14.1)	3.2 [1.9–5.5]
HADS-D (*N* = 109)	44 (40.4)	1.3 [0.7–2.4]	36 (33.0)	0.9 [0.5–1.9]	11 (10.1)	1.1 [0.5–2.8]
HADS-cAD (*N* = 137)	64 (46.7)	2.5 [1.2–4.8]	42 (30.7)	1.4 [0.7–2.9]	17 (12.4)	2.8 [1.2–6.5]

AOR: adjusted odds ratio (multivariate logistic regression analysis, using participants with no psychiatric disorder as reference and adjusted for age, gender, household consumption, habitation, altitude and use of alcohol and marijuana), d/m: days/month

Tables 6 and 7 show how the effects of comorbid headache and HADS caseness interacted on QoL, being additive (reducing WHOQOL score) in almost all cases. The only exceptions were that the presence or absence of TTH made little difference to QoL in those with HADS-A or HADS-cAD. In general, the additive effects of comorbid HADS caseness were highly significant; those of HADS-cAD were strongest and those of HADS-A least strong. None of the effects of the headache types were significant although, apart from those of TTH, they were consistent; headache on ≥15 days/month had, numerically, the strongest effect (Table 7).

**Table 6:** Quality of life (WHOQOL scores) among participants with headache, by headache type, with and without comorbid psychiatric disorder

Headache type	WHOQOL score (mean ± SD)								
	HADS-A caseness (*N* = 340)			HADS-D caseness (*N* = 109)			HADS-cAD caseness (*N* = 137)		
	Present	Absent	p*	Present	Absent	p*	Present	Absent	p*
Migraine (*N* = 728)	25.7 ± 4.1	26.9 ± 3.9	<0.001	24.6 ± 2.9	26.9 ± 3.9	<0.001	22.7 ± 3.6	27.1 ± 3.7	<0.001
Tension-type headache (*N* = 863)	27.2 ± 3.8	28.4 ± 3.7	<0.001	24.9 ± 3.2	28.4 ± 3.7	<0.001	24.4 ± 3.2	28.4 ± 3.6	<0.001
Headache on ≥15 d/m (*N* = 161)	25.1 ± 4.2	26.2 ± 3.9	0.10	24.0 ± 3.2	25.9 ± 4.1	0.11	21.6 ± 3.4	26.4 ± 3.8	<0.001

*Student’s t-test, d/m: days/month

**Table 7:** Quality of life (WHOQOL scores) among participants with HADS caseness, with and without comorbid headache

HADS caseness	WHOQOL score (mean ± SD)								
	Migraine (*N* = 728)			Tension-type headache (*N* = 863)			Headache on ≥15 d/m (*N* = 161)		
	Present	Absent	p*	Present	Absent	p*	Present	Absent	p*
HADS-A (*N* = 340)	25.7 ± 4.1	26.5 ± 2.8	0.30	27.2 ± 3.8	26.5 ± 2.8	0.36	25.1 ± 4.2	26.5 ± 2.8	0.096
HADS-D (*N* = 109)	24.6 ± 2.9	26.5 ± 3.9	0.055	24.9 ± 3.2	26.5 ± 3.9	0.15	24.0 ± 3.2	26.5 ± 3.9	0.094
HADS-cAD (*N* = 137)	22.7 ± 3.6	24.2 ± 3.2	0.21	24.4 ± 3.2	24.2 ± 3.2	0.86	21.6 ± 3.4	24.2 ± 3.2	0.060

*Student’s t-test, d/m: days/month

## Discussion

This is the first nationwide, population-based survey in any country of South Asia to explore the comorbidity of headache and psychiatric disorders, which are both highly prevalent in Nepal [31, 32]. Comorbidity occurred more than was expected by chance, at least with regard to migraine and headache on ≥15 days/month on the one hand, and HADS-A and HADS-cAD on the other, the associations thereby indicated (with AORs in the range 1.7–3.2) likely to be bidirectional. There were also significant associations between headache, especially headache occurring on ≥15 days/month, and neuroticism. There were no associations between any headache and HADS-D, and none between TTH and any HADS caseness. Additive and possibly synergistic effects of comorbid headache and HADS caseness were evident on QoL, highly significantly so when migraine or TTH was the index disorder and HADS caseness the comorbid disorder.

Before considering the implications of these findings, we would compare them with others as a test of veracity; but as we have noted, there are none from this Region. Outside South Asia, most studies exploring psychiatric associations with headache disorders have focused on migraine [3–11]. A Zürich cohort study first demonstrated strong relationships between migraine and both anxiety and depression [3], which were subsequently reported in the United States (US) [4, 9] and Canada [42]. Other US studies found that people with migraine had high levels of neuroticism [43, 44]. In Nepal, we found strong associations between migraine and HADS-A, HADS-cAD and neuroticism, but not HADS-D.

There is relatively little evidence regarding psychiatric associations with episodic TTH [45]. A large Swiss epidemiological study found no associations with depressive or anxiety disorders [7], and neither did we. Episodic TTH is usually a less painful disorder than migraine, and less troublesome since it lacks the range of associated symptoms that contribute to the burden of migraine [35]. Hence people with episodic TTH may not express the same extent of subjective emotional experiences as those with migraine [46].

People with chronic TTH, on the other hand, demonstrate strong psychiatric associations [47]: in the US, almost half had either a depressive or an anxiety disorder [48] while those in a Norwegian study had high levels of neuroticism [22]. We did not analyse chronic TTH specifically because it could not reliably be diagnosed by lay interviewers using a questionnaire [35], but among our participants with other headache on ≥15 days/month would have been some with this disorder. The category of headaches occurring on ≥15 days/month corresponds with what is elsewhere referred to as “CDH” [49], which has been associated with high frequencies of both depressive and anxiety symptoms [13, 50]. We found, in the Nepalese population, that headache on ≥15 days/month was associated with HADS-A, HADS-cAD and neuroticism, but not HADS-D.

Our findings, therefore, are not entirely in accord with others regarding depression. The explanation may be methodological. We found no association between HADS-D and any headache type, in line with French [27] and Norwegian studies [51] that also used HADS to establish psychiatric caseness. The studies from Switzerland [3], the US [4] and Canada [42], which found strong associations between depression and headache, instead used structured interviews based on Diagnostic and Statistical Manual (DSM) criteria [52]. A recent study from South Korea [53] obtained similar findings using the self-administered Patient Health Questionnnaire-9 (PHQ-9) [54], as did another US study [48] using the Beck Depression Inventory (BDI) [55]. Cultural factors determining how moods are expressed [56] may contribute: in many Asian countries, depression is often manifested somatically [57, 58], while HADS does not capture the somatic domains of depression so well [38]. HADS may therefore underestimate any association of depression *alone* with headache.

However, as was pointed out in a recent review [20] and revealed in the French study [27], depression in migraine rarely occurs alone but is almost always comorbid with anxiety. A stronger association of migraine with combined anxiety-depression than with either independently was seen in the early Zürich study [3], and similar findings in relation to the other headache types came later in Norway [51]. In our study, HADS-cAD occurred twice as often among all headache sufferers and those with migraine, and almost three times in those with headache on ≥15 days/month. Hence the association of headache with depression in our population, though not apparent in HADS-D, may have manifested in HADS-cAD. There was, however, a gender influence in this association: for any headache, the association with HADS-cAD appeared specific to females (AOR 4.3 *versus* 1.0 in males).

Summarising these arguments, we believe use of HADS – a screening rather than diagnostic instrument for depression – is valid among people with headache although it may underestimate caseness prevalence. We could not establish any association between headache disorders and HADS-D in Nepal, but this limitation should be taken into account.

We also note other limitations. It is a general limitation of cross-sectional studies that, while associations can be demonstrated, causality cannot. However, public-health purposes are served in the first instance by uncovering these relationships, which have important policy implications; subsequent research can investigate causation. It is a limitation of HADS that it cannot go beyond detecting caseness of anxiety or depression, which encompass heterogeneous mental disorders. This renders it difficult to identify and make provision in health-service planning for issues that might be related to particular types of these disorders. By a similar token, our headache diagnostic questionnaire captured episodic migraine and TTH, while all chronic cases were subsumed under headache on ≥15 days/month [35]. Comorbidities associated with these subtypes were likely to be different in view of their high-frequency, long-duration, intractable symptoms coupled with problems in management, but the prevalence of each would have been about 1 % or less. Categorically assessing these multiple types and subtypes would have been a cumbersome exercise requiring much greater investment. We believe that our approach, with simple validated interviewer-administered culturally-validated instruments, was a more practical option in a population with high illiteracy, and it best served our public-health purpose. Furthermore, our study had notable strengths: tried and tested methodology [33–35, 59], large sample size, high participation rate and good representativeness of the diverse population of Nepal [33, 34].

### Implications for Nepal

From the public-health perspective, there is reason for alarm in the high prevalences of these MDBs in Nepal. We found a clear negative impact on QoL in people with headache and comorbid psychiatric disorder. GBD2013 provides disability weights for these disorders [25], which, multiplied by prevalence, yield estimates of disability burden. Migraine, MOH, depression and anxiety are all in the top 20 causes of YLDs globally [25], while all seem to be more prevalent in Nepal than their respective global means [31, 32]. These facts, together with evidence of excessive comorbidity between headache disorders and anxiety, and indications of aggravated burden when they are comorbid, signal an urgent need for action backed by health policy. The global context is not good, either for headache, which is undertreated everywhere [60] (a failure that should not discourage attempts at remediation [26]), or for psychiatric disorders, for which only a minority of affected people receive adequate treatment in most countries around the world [61]. In Nepal, an underdeveloped country with multiple adversities [33], the challenges brought forward by these comorbid conditions are considerable; this resource-deficient country is not prepared to cope with them.

From the clinical perspective, patients with headache disorders can be expected to have an excess of psychiatric manifestations, and *vice versa*. Physicians in Nepal treating headache patients should be looking out among them for anxiety and depression as potentially aggravating comorbid factors likely to hinder management. Moreover, psychiatrists and others treating depression and anxiety can expect to encounter migraine and headache on ≥15 days/month at high levels. We summarise this as a need for reciprocal awareness.

We propose that a coordinated effort offers a solution from both perspectives – public-health and clinical. It requires training of health-care providers encountering headache patients to think beyond the somatic dimension: screening for both anxiety and depression, and collaborating with mental-health personnel in patient education on lifestyle, psychological treatment and behavioural strategies, may be appropriate options in addition to offering usual pharmacological interventions. This already happens to an extent in some cities where headache patients may be referred directly to psychiatrists, but these are a small minority. In the rural areas and in the high hills, such referrals are generally not possible. In these areas, and in the cities also, most headache care is and should be provided in primary care, for reasons related both to logistics and cost [60], as well as because it is feasible. In primary care is also where most depression and anxiety are encountered [62]. Bringing the management of common headache and common psychiatric disorders together under one primary-care roof, supported by educational initiatives and referral channels to specialist services, appears to be good health policy. We recommended its trial implementation in a circumscribed area as a first step, and enter a plea for urgency in this action.

## Conclusion

Headache disorders, highly prevalent in Nepal, are excessively comorbid with anxiety and associated with neuroticism, and these relationships aggravate their negative impact on QoL. These findings call for reciprocal awareness, and a holistic coordinated approach to management and in the health service. Care for common headache and common psychiatric disorders should be integrated in primary care.

## Abbreviations

AOR: adjusted odds ratio
BDI: Beck Depression Inventory
cAD: comorbid anxiety and depression
CI: confidence interval
CMD: common mental disorder
CDH: chronic daily headache
DSM: Diagnostic and Statistical manual of Mental disorders
EPQRS-N: Eysenck Personality Questionnaire Revised Short Form-Neuroticism
GBD: Global Burden of Disease
HADS: Hospital Anxiety and Depression Scale
HARDSHIP: Headache-Attributed Restriction Disability, Social Handicap and Impaired Participation
ICD: International Classification of Diseases
ICHD: International Classification of Headache Disorders
LAMI: low-and-middle-income
MDB: major disorders of the brain
NTNU: Norwegian University of Science and Technology
OR: odds ratio
PHQ: Patient Health Questionnaire
pMOH: probable medication-overuse headache
QoL: quality of life
SPSS: Statistical Package for Social Science
TTH: tension-type headache
US: United States
WHOQOL-8: World Health Organization Quality-of-Life 8-question scale
YLD: year of life lost to disability
